# Using the Doubly Charged Selected Ion Coupled with MS/MS Fragments Monitoring (DCSI-MS/MS) Mode for the Identification of Gelatin Species

**DOI:** 10.1155/2014/764397

**Published:** 2014-03-13

**Authors:** Xian-Long Cheng, Feng Wei, Jia Chen, Ming-hua Li, Lei Zhang, Ying-Yong Zhao, Xin-Yue Xiao, Shuang-cheng Ma, Rui-Chao Lin

**Affiliations:** ^1^School of Chinese Pharmacy, Beijing University of Chinese Medicine, No. 6 Wangjing Zhong Huan Nan Lu, Chaoyang District, Beijing 100102, China; ^2^Institute for the Control of Traditional Chinese Medicine and Ethnic Medicine, National Institutes for Food and Drug Control, State Food and Drug Administration, 2 Tiantan Xili, Beijing 100050, China; ^3^Key Laboratory of Resource Biology and Biotechnology in Western China, Ministry of Education, The College of Life Sciences, Northwest University, No. 229 Taibai North Road, Xi'an, Shaanxi 710069, China

## Abstract

In electrospray ionization (ESI) mode, peptides and proteins can be multiply charged ions; in this situation a doubly charged selected ion (DCSI) coupled with mass spectrometry (MS/MS) fragments monitoring (DCSI-MS/MS) method is the most suitable scanning mode to detect known peptides in complex samples when an ion-trap mass spectrometer is the instrument used for the analysis. In this mode, the MS detector is programmed to only select a doubly charged ion as a precursor and to perform continuous MS/MS on one or more of the selected precursors, either during a specific time interval or along the whole chromatographic run. Gelatin is a mixture of high molecular weight polypeptides from the hydrolysis of collagen. In this study, the DCSI-MS/MS monitoring mode was applied to the detection of previously characterized species-specific peptides from different gelatins. The proposed methodology makes use of tryptic digestion for sample preparation and peptide separation and identification by rapid resolution liquid chromatography coupled to an ion trap working in the DCSI-MS/MS mode for the analysis. This methodology was applied to the differential classification of five commercial, homological species of gelatins and proved to be an excellent tool for gelatin product authentication.

## 1. Introduction

The assessment of gelatin authentication and origin is of major concern for standards authorities, not only to assist in the prevention of commercial fraud in the food, cosmetic, and pharmaceutical industries [1, 2], but also to help avoid the safety risks derived from diseases among livestock that might be harmful for human health. The correct identification of gelatin species thus becomes an issue of primary relevance for these industries.

The characterization of external morphological features is a particularly difficult method for gelatin species differentiation because of their apparent similarities and to the fact that they are frequently lost during the manufacturing process. An alternative, molecular method for gelatin species identification based on DNA or protein analysis is tedious and time consuming. Polymerase chain reaction (PCR) method has been used in DNA analysis but again is not available for gelatin identification because of the heavy destruction of DNA in gelatin during processing, although this method has been widely applied in collagen identification [3, 4]. Similarly, the immunochemical method has been used to identify collagen [5], but again the usefulness of this method might be influenced by the extent of the proline hydroxylation which plays an important role in determining the antigenicity of collagen. As a result of the problems with the methods that we have outlined, proteomics methods have been proposed as alternative tools for the assessment of the authenticity and traceability of collagen species in gelatins [6], and mass spectrometry has been successfully applied to elucidate differences among homological gelatins [7]. In line with these results, we developed an analytical method based on proteomics to distinguish these gelatins effectively. This method will also prevent the possibility of mislabeling and adulterations.

Collagens from different sources contain differential amino-acidic sequences. In previous studies [8], we have characterized the specific peptides from five species of gelatins by means of UPLC-QTOF/MS coupled with PCA. Those specific peptides can be used as specific markers for gelatin product authentication.

The analysis of tryptic peptides, as commonly used in proteomics, is a powerful technique for the identification of proteins [9, 10]. A complex peptide sample is typically first separated into its constituents by using HPLC-DAD (diode array detector) and HPLC/MS/MS [11, 12] procedures. In recent years, the new analytical technique of ultraperformance liquid chromatography has been developed for the study of proteins [13, 14]. This method uses small particle size packing in the column to provide better chromatographic resolution.

A peptide resulting from tryptic digestion normally has a basic residue (arginine or lysine) at its C-terminus and yields a prominent doubly charged ion peak when ionized by ESI. If this ion is chosen as the parent ion for an MS/MS measurement, the production of a series of y-ion daughters (ions resulting from cleavage at the amide bonds and containing the C-terminus) is favored and the resulting spectrum is likely to be easy to interpret [15]. For this reason we selected the doubly charged ion as the parent ion for our analysis.

The objective of this work is the study of gelatins using the doubly charged selected ion coupled with MS/MS fragments monitoring (DCSI-MS/MS) to aid in the identification of gelatins. The gelatins were digested by trypsin, liquid chromatography separation, and peptide identification by MS using the DCSI-MS/MS scanning mode, as a reliable method for fast and effective gelatin identification. In addition, the possibility of detecting the target peptides in these samples with rapid resolution liquid chromatography (RRLC) separation, using an electrospray ionization- (ESI-) ion trap (IT) MS, was tested.

## 2. Experimental

### 2.1. Chemicals

Formic acid was purchased from Sigma-Aldrich (St. Louis, MO, USA). Acetonitrile (HPLC grade) was purchased from Merck (Rahway, NJ, USA). Methanol (HPLC grade) was purchased from Fisher Scientific (Pittsburgh, PA, USA). Ultra-high-purity water was prepared by a Milli-Q water purification system (Millipore Corporation, Bedford, Massachusetts). Trypsin (sequencing grade) was obtained from Promega (Madison, WI, USA). A syringe filter (0.22 *μ*m) was purchased from Millipore (Billerica, MA, USA). All other chemicals used were of analytical grade. Five different gelatins were made by lab for analysis. Five of the 20 samples tested were donkey-hide gelatins, belonging to the family Equidae, six samples were bovine-hide gelatins belonging to the family Bovidae, three samples were porcine-hide gelatins belonging to the family Suidae, three samples were deerhorn gelatins belonging to the family Cervidae, and three samples were of Tortoise shell glue, belonging to the family Emydidae. Twenty commercial samples were collected on market.

### 2.2. Peptide Sample Preparation

First, 100 mg of the gelatin standard was dissolved in 50 mL of a 1% NH_4_HCO_3_ solution (pH 8.0). The solution was filtered through a 0.22 *μ*m syringe filter. Then, 100 *μ*L of the gelatin solution was drawn, and 10 *μ*L of trypsin solution (1 mg/mL in 1% NH_4_HCO_3_, pH 8.0) was added. The mixture was incubated at 37°C for 12 hours.

## 3. Equipment

Chromatographic separation was performed on an Agilent G6320 series LC/MSD Trap Mass Spectrometer system. The actual chromatographic separation was carried out using an Agilent 1200 Series Rapid Resolution LC system (Agilent Technologies, USA), equipped with a binary pump, a microvacuum degasser, a high performance autosampler, a column compartment, a diode array detector, and a MS detector. The samples were separated on an Agilent Zorbax SB-C8 (100 mm × 2.1 mm, 1.8 *μ*m). The mobile phase consisted of solvent A (0.1% formic acid in water, v/v) and solvent B (acetonitrile). The optimized RRLC elution conditions were as follows: 0.0–25.0 min, 5%–20% B; 25.0–40.0 min, 20–50% B; 40.0–41.0 min, 50–99% B; 40.0–45.0 min, 99% B; 45.0–45.1 min, 99.0–5.0% B; 45.1–55.0 min, 5.0%. The flow rate was set at 300 *μ*L/min. The column and autosampler were maintained at 40°C and 10°C, respectively. The injection volume was 5 *μ*L.

Mass spectrometry experiments were performed with the ESI source in positive ion mode. The vaporizer temperature was maintained at 350°C. The temperature of the drying gas was set at 350°C. The flow rate of the drying gas and the pressure of the nebulizing gas were set at 6 L/min and 60 psi, respectively. The capillary voltage was kept at 3.5 × 10^3^ V. Full-scan spectra were acquired over a scan range of* m/z* 50–2200. Qualitative analyses were programmed to be carried out by using the doubly charged selected ion monitoring (DC-SIM) mode of the doubly charged ion peak from previously characterized peptides [12]. An Agilent ChemStation was used to control and process the data from the Agilent 6320 Series Ion Trap LC-MS. 

## 4. Results and Discussion

### 4.1. Specificity

Specificity was performed by using one gelatin as a sample, while the other four gelatins were present as blank samples. The chromatographic peak was verified by comparing the retention times and the fragments of the peaks, with the retention times and the fragments of the characterized species-specific peptides of every kind of gelatin listed in Table 1.

### 4.2. Repeatability

**Figure 1:**
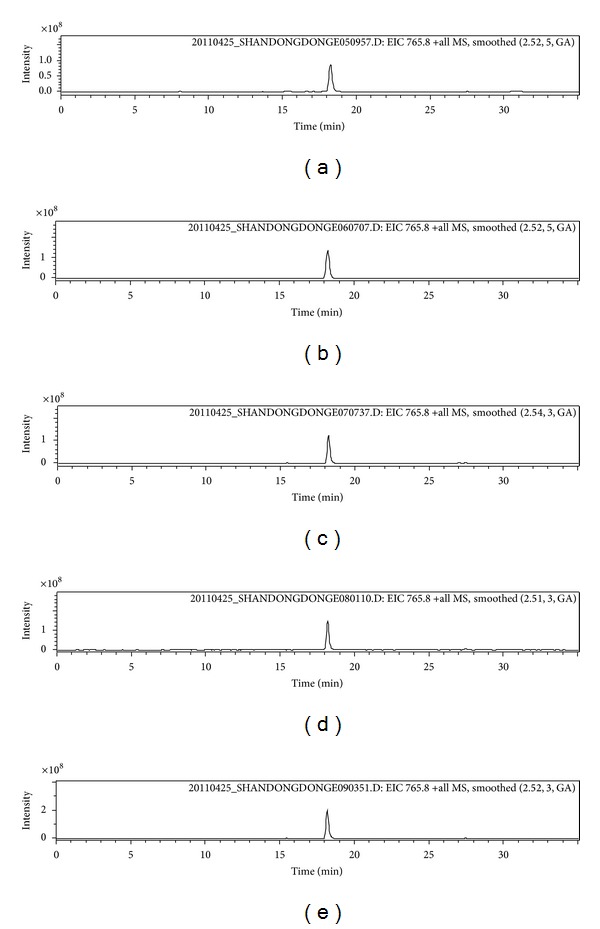
Doubly charged selected ion chromatograms obtained from the tryptic digests analysis of five donkey-hide gelatins.

Five same samples were digested in the same condition; the marker peptides in these five same samples could be detected; the selected ions chromatograms for the five samples from the family Equidae are shown in Figure 1.

### 4.3. Stability

The freeze and thaw stability of tryptic samples was detected. The samples were frozen at −20°C, thawed at room temperature, and then, when completely thawed, refrozen for 24 hours under the same conditions with at least a 12-hour interval between each cycle. This freeze-thaw cycle was repeated three times before the analysis of the samples was carried out using the method described above. Long-term stabilities were measured by leaving samples at −20°C or at ambient temperature for a certain time period (20 days or 24 hours, resp.), after digestion. The results show that the characterized species-specific peptides of samples kept at −20°C for 20 days and then thawed at room temperature can still be detected.

### 4.4. Species Identification by DCSI-MS/MS in Gelatins

**Figure 2:**
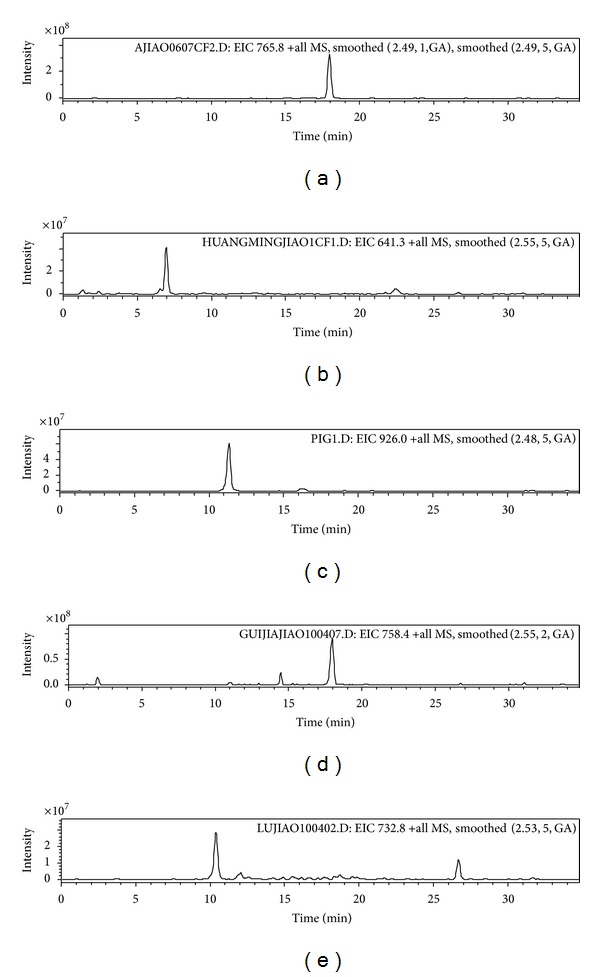
Doubly charged selected ion chromatograms obtained from the tryptic digest analysis of (a) donkey-hide gelatin, (b) bovine-hide gelatin, (c) pig-hide gelatin, (d) tortoise shell glue, and (e) deerhorn glue.

**Figure 3:**
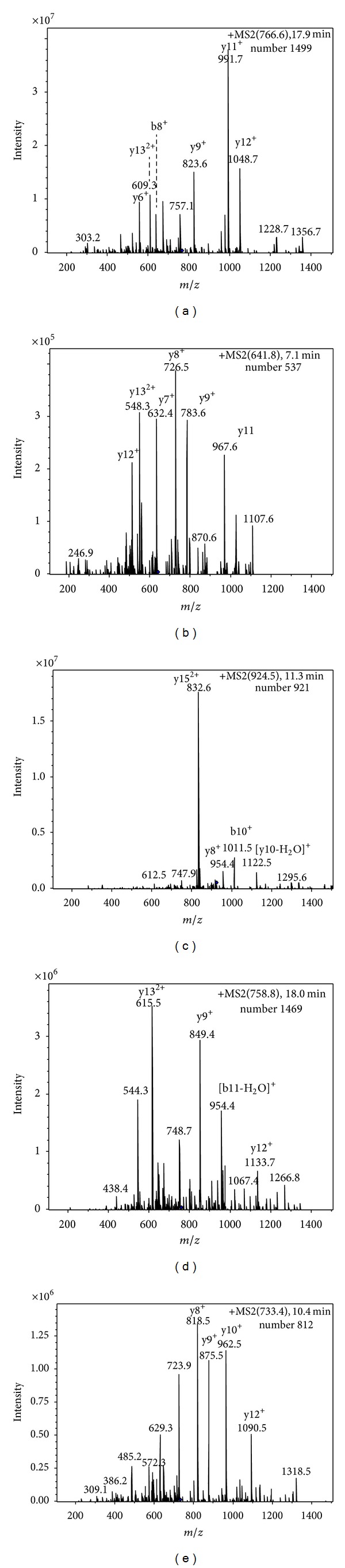
MS/MS fragmentation spectra of the ions at* m/z* (a) 765.8, (b) 641.8, (c) 924.5, (d) 758.8, and (e) 732.8 obtained by DCSI-MS/MS analysis, showing the fragment peaks that matched the expected peptide sequences. Gelatins were digested with trypsin.

The complex peptide pools obtained by tryptic digestion of gelatins were subjected to LC-MS/MS, analyzing only five precursor ions at* m/z* 765.8,* m/z* 641.8,* m/z* 924.5,* m/z* 758.8, and* m/z* 732.8, which were the doubly charged ions of the previously described species-specific peptides [12]. Once the MS/MS spectra of these precursor ions were recorded, chromatogram traces for the different fragment ions could be obtained. The fragmented ions produced by precursor ions were determined by the sequence of the marker peptide, so the averaged MS/MS spectra obtained around this retention time gave a perfect agreement with the peptide pattern, even without knowledge of sequence of the marker peptides.  Figure 2 shows the selected ion chromatography of five gelatins obtained by DC-SMIM.  Figure 3(a) is the doubly charged ion* m/z* 765.8 fragmentation spectra representing the presence of the characteristic species-specific peptide of donkey-hide gelatin. Four spectra proving the presence of the corresponding characteristic species-specific peptides for the remaining gelatins are shown in Figures 3(b)–3(e).

Commercial samples were also effectively identified after matching specific peptides in these samples with the corresponding reference samples. Ten of twenty commercial samples were donkey-hide gelatins, five were bovine-hide gelatins, three were deerhorn gelatins, and two were Tortoise shell glue. This method enables the researcher to identify the exact species correctly and efficiently.

## 5. Conclusions

The results presented in this paper confirm the validity of our previously presented approach, which showed that the characterized species-specific peptides of gelatins can be used to distinguish one gelatin from a second or to identify individual gelatins in a mixture. In our work, five doubly charged ions at* m/z* 765.8,* m/z* 641.3,* m/z* 924.5,* m/z* 758.8, and* m/z* 732.8, which are the respective species-specific peptides of donkey-hide gelatin, bovine-hide gelatin, pig-hide gelatin, tortoise shell glue, and deer-horn glue, were selected as monitor ions. Our results show that using the method we outline in this paper based on the high separation capability of RRLC and the peptide identification ability of MS using the DCSI-MS/MS scanning mode to detect and monitor diagnostic peptides from five different gelatins is simple, rapid, and exclusive. The method is shown to be suitable for identifying five gelatins in a mixture.

## Figures and Tables

**Table 1:** Retention times and fragments of characterized species-specific peptides from each gelatin.

*m*/*z* observed	Retention times	Fragments	Sequence	Originated from
765.8	17.9	1084.7, 991.7, 823.6, 609.3	GEAGPAGPAGPIGPVGAR	Donkey-hide gelatin
641.8	7.1	1107.6, 967.6, 783.6, 726.5, 632.4, 548.3	GEAGPSGPAGPTGAR	Bovine-hide gelatin
924.5	11.3	1122.5, 1011.5, 832.6, 747.9,	GEPGPTGVQGPPGPAGEEGK	Pig-hide gelatin
758.8	18.0	1133.7, 954.4, 849.4, 748.7, 615.5, 544.3	GDGGPP(OH)GITGFPGASGR	Glue of tortoise shell
733.4	10.4	1090.5, 962.5, 875.5, 818.5, 723.9, 629.3	SGETGASGPP(OH)GFAGEK	Deerhorn glue
